# Tuberculosis (TB) in the refugee camps in Ethiopia: trends of case notification, profile, and treatment outcomes, 2014 to 2017

**DOI:** 10.1186/s12879-021-05828-y

**Published:** 2021-02-03

**Authors:** Tsegay Legesse, Goitom Admenur, Selemawit Gebregzabher, Eyob Woldegebriel, Bexabeh Fantahun, Yemane Tsegay, Abeyot Bayssa, Berihu Darge, Yidnekachew Denbu, Hayelom Michalel, Kibebew Abera, Abraham Alemayeh, Dejene Kebede, Desta Kasa

**Affiliations:** 1 Inter-Governmental Authority on Development (IGAD), Djibouti, Djibouti; 2 Administration for Refugee and Returnee Affairs, Addis Ababa, Ethiopia; 3 Federal Ministry of Health, Addis Ababa, Ethiopia; 4 United Nations Higher Commision for Refugees, Addis Ababa, Ethiopia

**Keywords:** Tuberculosis, Refugees, Refugee camps, Case notification, Treatment outcome

## Abstract

**Background:**

Severity of TB increases in refugee populations. Monitoring TB case notification and treatment outcomes are essential to improve the effectiveness of TB programs. This study aimed to investigate trends in TB case notification and treatment outcomes and explore factors associated with unsuccessful treatment outcome in refugee camps in Ethiopia.

**Methods:**

In this retrospective cohort study, demographic and clinical data of all TB cases registered in 25 refugee camps in Ethiopia from January 2014 to December 2017 were extracted. Multivariate logistic regression was fitted to estimate odds ratios and corresponding 95% confidence intervals for the measure of association linked with factors significantly associated with unsuccessful treatment outcomes.

**Results:**

A total of 1553 TB cases, mean age 27.7 years, were registered from 2014 to 2017. Of these notified cases 54.7% were men, 27.7% children (< 15 years), 71.2% pulmonary TB (PTB), 27.8% Extra-PTB (EPTB) and 98.3% new and relapse. From 2014 to 2017: there was consistent increase in number of notified TB cases (138 to 588 cases), in percentage of EPTB (23.2 to 32.7%), in contribution of children to total TB cases (18.8 to 30.1%) and to EPTB (40.6 to 65.1%), and in proportion of bacteriologically confirmed new and relapse pulmonary cases (43.8 to 64.8%). Treatment success rates for all TB cases remained lower at 72.7–79.4%. On average 24.8% had unfavorable treatment outcome, including 11.5% not evaluated, 8.0% LTFU, 4.8% died and 0.5% treatment failed. Unsuccessful treatment was significantly associated with pretreatment weight below 40 Kg, age over 45 years, and being HIV positive.

**Conclusions:**

There was continuous increase in notified TB cases and in percentage of childhood TB. Proportion of bacteriologically confirmed new and relapse pulmonary cases increased overtime. TB treatment success remained lower than the national achievement in 2017 (96%) and global target (> = 90%), which needs improvement. The higher LTFU, not evaluated, and death suggests the need to strengthen adherence education and supervision. Special socio-economic support and monitoring is required for patients with pretreatment weight below 40 Kg, age over 45 years and HIV positives.

**Supplementary Information:**

The online version contains supplementary material available at 10.1186/s12879-021-05828-y.

## Background

Tuberculosis (TB) is an ancient infectious disease caused primarily by *Mycobacterium tuberculosis* [1]. Despite that TB burden has been declined in the past 20 years in the globe, currently TB is the leading cause of death from single infectious agent above HIV/AIDS. Globally in 2018, there were an estimated 10 million incident TB cases, 1.5 million deaths from TB, and 0.5 million people with drug resistant TB. In addition, 8.6% of TB cases in the world were living with HIV, of whom 72% were in Africa [2].

TB is most sever in migrants, refugees and displaced populations (key populations) due to reasons including poor shelter and living conditions, poor health and nutritional status, overcrowding, and inadequate access to TB care and prevention [3–6]. Thus, these key populations face higher risk to TB infection and diseases progression [3–6], contracting or developing MDR-TB [7] and unsuccessful TB treatment outcome [8]. Reports showed lower treatment success rate in TB patients among refugee populations (74.2%) compared to surrounding communities (88.1%) in Gambella region in Ethiopia [8]. Therefore, in order to end TB by 2030, strengthen TB care and prevention in refugee and displaced population has been listed as one of the 10 components of the End TB strategy by world health organization (WHO) [2].

Overall, armed conflicts and population displacements associated with up to 20-fold increases in the risk of TB [6]. In 2018, there were 70.8 million refugees, asylum seekers and persons displaced by wars and conflicts worldwide [9]. Usually, majority (> 85%) of refugees originate from and remain within countries with high burdens of TB [10]. However, according to a recent systemic review and meta-analysis, TB prevalence in refugees and asylum seekers varied more according to countries of origin than the host continent [11]. Other also reported, an association between immigration and increased prevalence of either TB or EPTB [12]. Thus, following the continuous immigration of Syrian refugees, TB case notification has increased in Syrian refugees in Lebanon from 2010 (8 cases) to 2016 (147 cases) [13], and cased detection rate/100000 population increased in the Syrian refugee in Jordan by 40% from 2013 (0.73 cases) to 2015 (1.01 cases) [14].

Ethiopia, a country with population size of 109 million, is among the 30 high burden countries for TB, TB/HIV and MDR-TB. By 2018, the estimated total TB incident, notified TB cases and TB mortality in Ethiopia was 165,000, 114,233 and 24,000, respectively [2]. Nonetheless, according to United Nations High Commission for Refugees (UNHCR) [15], the number of refugees in Ethiopia (hosted in 26 refugee camps included in this study) continuously increased from 491,030 refugees in 2014, to 587,790 in 2015, 653,063 in 2016 and then peaked to 728,113 refugees by 2017. Majority of the refugees originated from countries TB is predominant which are South Sudan, Somalia, Eritrea and Sudan [15].

In summary, taking into account the high TB burden in the host country (Ethiopia) and in the country of origin of the refugees [2], the large refugee population in Ethiopia which increased overtime [15], and the higher risk of the refugee population to TB infection and disease progression [3–6], to MDR-TB [7] and to unsuccessful TB treatment success [8, 16], TB can be major health problem in the refugee camps in Ethiopia. Therefore, in order to improve the effectiveness of TB control and prevention in the refugee camps, the performance of the TB programs need to be evaluated and monitored regularly. Among the main indicators for TB program performance are TB case finding, notification, and treatment outcome [17, 18]. The aim of this study was to investigate trends on TB case notification, profile, and treatment outcome; and to explore factors associated with unsuccessful TB treatment outcomes in the refugee camps in Ethiopia from 2014 to 2017.

## Materials and methods

### Study settings

Ethiopian has a three-tier health care delivery system (a primary health care tier comprising a primary hospital, health centers and health posts, a General Hospital tier, and a Specialized Hospital tier). The health facilities in the national health care delivery system undertake TB and leprosy prevention and control activities at their level. Thus, more than 3000 health facility in nine regional states and two administrative cities in Ethiopia deliver TB diagnosis and treatment services. The national TB program (NTP) under the Federal Ministry of health (FMOH) coordinate, support and lead the TB programs in the country.

In Ethiopia, there are 26 refugee camps located in six regional states namely Tigray, Afar, Gambella, Benishangul, Somalia and Southern Nations and Nationalities Peoples Region (SNNPR). In each refugee camp, there is health facility which deliver general health services including TB control and prevention. The TB programs in the refugee camps, are supported and lead by the (NTP) and adhere to the National guidelines for TB, DR-TB and Leprosy of Ethiopia [17].

The 26 refugee camps in Ethiopia are sub-organized under seven refugee areas namely Shire, Afar, Gambella, Asossa, Mizan, Jijiga and Dollo Ado: 1) Shire refugee area: found north west of the country in Tigray region, hosts four refugee camps, 2) Afar refugee area: found east of the country in Afar region, hosts two refugee camps, 3) Gambella refugee area: found west of the country in Gambella Region, hosts six refugee camps, 4) Mizan refugee area: found south of the country in SNNPR, hosts one refugee camps, 5) Asossa refugee area: found west of the country in Benishangul region, hosts four refugee camps, and 6) Jijiga and 7) Dollo Ado refugee areas, found east of the country in Somalia region, hosts three and five refugee camps, respectively.

The total end year refugee population in Ethiopia was continuously increased from 491,030 in 2014 to 728,000 by 2017. The highest refugee population by 2017 were in Gambella refugee area (219,708), followed by Dollo Ado (197.952), Shire (109, 360), Asossa (55.726), Jijiga (35,024), Afar (15.390), and Mizan (11,761) [15].

Based on the inclusion critrea (heatlh facilites which have both TB diagnosis and treatment servces starting at least in January 2017), this study was conducted in 25 refugee camps/25 health facilities (HFs) located in seven refugee areas (4 HFs in Shire, 2 HFs in Afar, 6HFs in Gambella, 1 HF in Mizan, 4 HFs in Asossa, 3 HFs in Jijiga, and 5 HFs in Dollo Ado) (Supplement 1). All health facilities included in the study had standardized unit TB registers. One health facility out of the 26 which did not have TB treatment service since 2017 is excluded from the study.

### Study design, population, and data collection

The data collection for this 4 year (2014–2017) health facility based retrospective cohort study was conducted from January 2019 to April 2019. The study population were all refugee TB patietns registered from 2014 to 2017 in the 25 refugee health facilities. For each study participant, demographic and clinical data were extracted from Unit TB patient register by trained health officers and nurses using a pre-tested data collection Form. The data collection Form was pre-tested during the 4 day training we gave to the data collectors, supervisors and coordinators. For this, a previously filled and archived Unit TB register in the refugee health facilities were collected, and hands on training was given on data extraction from the Unit TB register and filling the data collection Form to each trainee. Finally, the data collection Form was revised as per the suggestion and comments received and approved.

Overall, to ensure data quality of this study, training was given to data collectors, study coordinators and supervisors; supervision was done on daily basis by field supervisors and weekly by national coordinators during data collection; and then 10% of the collected data were randomly selected by the study coordinators and were re-collected by the field supervisors and were checked page by page at the end of data collection.

### Data entry and statistical analysis

Data were coded and double entered into Epi-info version 7 by two trained data clerks and then cross-checked for consistency. Data were exported to STATA version 13 (Stata Corp, College Station, TX, USA) for data checking, cleaning, and analysis. During the preliminary analysis we looked for errors and corrected them by re-checking the data collection Form. Binary logistic regression analysis was done to identify independent variables associated with unsuccessful treatment outcome. Finally, multivariate logistic regression analysis was used to measure the independent effects of each predictor variable on unsuccessful treatment outcomes. Variables with a *P*-value of < 0.05 in the bivariate analysis were included in the multivariate model. Odds ratios with 95% confidence interval (CI) were used to assess the strength of association between variables. The independent variables used were age, sex, baseline weight, type of TB, category of TB patient, HIV infection and ART status. Statistical significance level was considered at a P-value < 0.05.

#### Definitions

As shown in Supplement 2, TB case definition and TB treatment outcomes were defined according to the standard definitions in the National guidelines for TB, DR-TB and Leprosy in Ethiopia [17] and WHO guideline [18].

## Results

### Characteristics of the study participants

The clinical and sociodemographic characteristics of the TB cases is shown in Table 1**.** From 2014 to 2017, a total of 1553 TB cases were registered in 25 refugee camps. The mean age of the total TB cases was 27.2 years, 430 (27.7%) were children under 15 years, 998(52.4%) were adults 15–44 years, 850 (54.7%) were male, and 690 (44.4%) were female.

**Table 1:** Sociodemographic and clinical characteristics of the notified TB cases (*n* = 1553) among the refugee camps in Ethiopia, 2014–2017

Characteristic	2014, N (%)	2015, N (%)	2016, N (%)	2017, N (%)	Total N (%)
**Total TB cases of all Forms**	138	354	473	588	1553
**Age group**					
< 15	26 (18.8)	92 (26.0)	135 (28.5)	177 (30.1)	430 (27.7)
15–29	50 (36.2)	107 (30.2)	141 (29.8)	152 (25.9)	450 (29.0)
30–44	39 (28.3)	87 (24.6)	107 (22.6)	132 (22.4)	365 (23.5)
45–59	15 (10.9)	43 (12.1)	46 (9.7)	79 (13.4)	183 (11.8)
> = 60	6 (4.3)	23 (6.5)	39 (8.3)	44 (7.5)	112 (7.2)
Not documented	3 (2.2)	2 (0.6)	4 (1.0)	4 (0.7)	13 (0.8)
**Mean age, years**	28.0	27.1	26.8	27.0	27.2
**Sex**					
Male	80 (58.0)	193 (54.5)	247 (52.2)	330 (56.1)	850 (54.7)
Female	57 (41.3)	160 (45.2)	222 (46.9)	251 (42.7)	690 (44.4)
Not documented	1 (0.7)	1 (0.3)	4 (0.9)	7 (1.2)	13 (0.8)
**Type of TB**					
PTB	106 (76.8)	279 (78.8)	334 (70.6)	389 (66.2)	1108 (71.3)
EPTB	32 (23.2)	73 (20.6)	134 (28.3)	192 (32.7)	431 (27.8)
Not documented	0	2 (0.6)	5 (1.1)	7 (1.1)	14 (0.9)
**Type of TB by diagnostic category**					
PTB+	47 (34.0)	162 (45.8)	216 (45.7)	252 (42.9)	677 (43.6)
Clinically diagnosed PTB^a^	59 (42.8)	117 (33.1)	118 (25.0)	137 (23.3)	431 (27.7)
EPTB	32 (23.2)	73 (20.6)	134 (28.3)	192 (32.6)	431 (27.8)
Not documented	0	2 (0.5)	5 (1.0)	7 (1.2)	14 (0.9)
**TB patient by previous treatment history:**					
New and relapse^b^	137 (99.3)	347 (98.0)	462 (97.7)	581 (98.8)	1527 (98.3)
Treatment after Failure	0	0	1 (0.2)	1 (0.2)	2 (0.1)
Lost to Follow up to Treatment	1 (0.7)	3 (0.8)	5 (1.1)	4 (0.6)	13 (0.8)
Others	0	1 (0.3)	2 (0.4)	1 (0.2)	4 (0.3)
Not documented	0	3 (0.8)	3 (0.6)	1 (0.2)	7 (0.5)
**New and relapse PTB patients:**	105 **(76.6)**	276 **(80.4)**	328 **(71.0)**	384 **(66.1)**	**1093 (71.6)**
*Bacteriologically confirmed cases:*	46 (43.8)	161 (58.3)	210 (64.0)	249 (64.8)	666 (60.9)
*Clinically diagnosed cases:*	59 (56.2)	115 (41.7)	118 (36.0)	135 (35.2)	427 (39.1)
**HIV status**					
Positive	10 (8.6)	48 (15.3)	50 (13.2)	57 (10.9)	165 (10.6)
Negative	106 (91.4)	265 (84.7)	329 (86.8)	464 (89.1)	1164 (75.0)
Not documented	22 (15.9)	41 (11.6)	94 (26.6)	67 (11.4)	224 (14.4)
**ART initiation**					
Yes	4 (40.0)	44 (91.7)	38 (76.0)	48 (84.2)	134 (81.2)
No	6 (60.0)	4 (8.3)	12 (24.0)	9 (15.8)	31 (18.8)
**TB cases by refugee areas**					
Shire	21 (15.2)	28 (7.9)	26 (5.5)	28 (4.8)	103 (6.6)
Afar	0	0	6 (1.3)	47 (8.0)	53 (3.4)
Gambella	43 (31.2)	159 (44.9)	288 (60.9)	368 (62.6)	858 (55.2)
Mizan	2 (1.4)	30 (8.5)	18 (3.8)	29 (4.9)	79 (5.1)
Asossa	14 (10.1)	29 (8.2)	16 (3.4)	14 (2.4)	73 (4.7)
Jijiga	45 (32.6)	42 (11.9)	45 (9.5)	34 (5.8)	166 (10.7)
Dollo Ado	13 (9.4)	66 (18.6)	74 (15.6)	68 (11.6)	221 (14.2)

*PTB+* Smear-positive pulmonary TB

^a^Clinically diagnosed PTB: includes smear-negative pulmonary TB and pulmonary smear unknown/not done

^b^New and relapse: includes cases for which the treatment history is unknown (not recorded) and transfer in; and it excludes cases that have been re-registered as treatment after failure, as treatment after lost to follow up or as other previously treated with unknown or undocumented treatment outcome

Among the total TB cases notifed, 1108 cases (72.0%) were PTB, 431 (28.0%) EPTB, and1527 (98.8%) new and relapse cases. Among the PTB cases, 677 (61.1%) were smear positive (PTB+), and 431 (38.9%) were clinically diagnosed (pulmonary smear negative plus smear unknown/not done). Among the 1527 new and relapse cases, 1093 (70.4%) were pulmonary cases, of whom 666 (60.9%) were bacteriologically confirmed and 427 (39.1%) were clinically diagnosed.

Of the total 1553 TB cases notified, 858 (55.2%) were from Gambella refugee area and 221 (14.2%) were from Dollo Addo. The remaining Afar, Asossa, Mizan, and Shire refugee areas contributed 3.4, 4.7, 5.1 and 6.6% of the total TB cases notified, respectively (Table 1). The number of notified TB cases for each refugee area and refugee health facility/refugee camp are presented in Supplement 1.

### Overall trends in TB case notification, 2014 to 2017

#### Trends in total case notification, by type of TB and treatment history

**Fig. 1:**
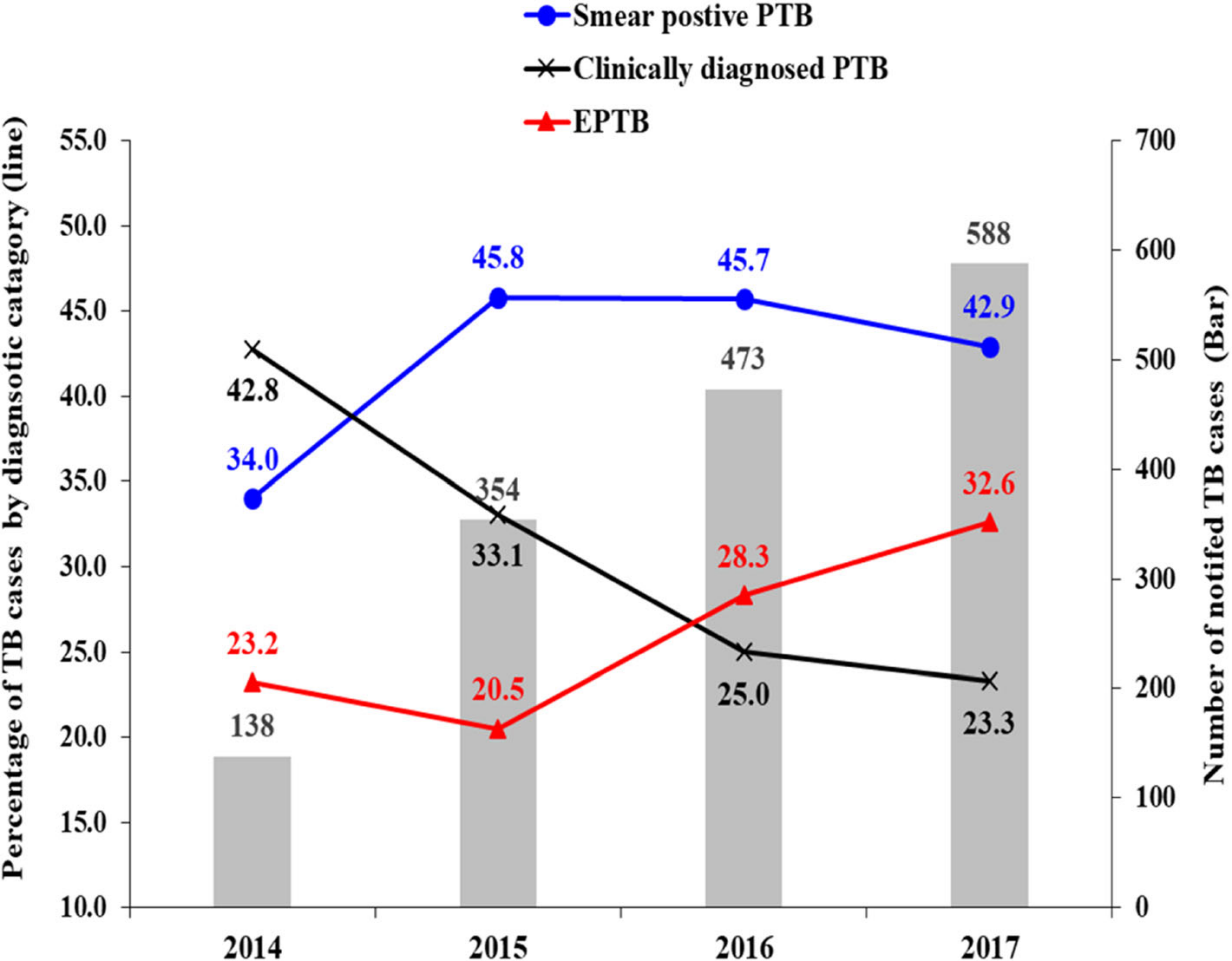
Trends in notified TB cases (bar) by diagnostic category (line) in the refugee camps, Ethiopia, 2014–2017

From 2014 to 2017: the number of notified TB cases of all forms increased from 138 to 588 cases. Among the notified cases, the percentage of EPTB increased from 23.2 to 32.7%, while PTB decreased from 76.8 to 66.2%. Among the PTB cases, the percentage of smear-positive pulmonary TB (PTB+) increased from 34.3% in 2014 to 42.9% % in 2017, and that of clinically diagnosed PTB (smear-negative pulmonary TB plus pulmonary smear unknown/not done) continuously declined from 42.8% in 2014 to 23.3% in 2017 (Table 1, Fig. 1). Among the pulmonary new and relapse cases, those bacteriologically confirmed increased from 43.8% in 2014 to 64.8% in 2017, and those clinically diagnosed declined from 56.2 to 35.2% (Table 1).

By treatment history, 97.7–99.0% of the TB patients registered during the study period were new and relapse; whereas on average 0.1, 0.8, 0.3 and 0.5%, respectively, were treatment after failure, lost to follow up to treatment, and “others” (Table 1).

#### Trends in cases notification stratified by gender

There was disparity in the notified TB cases by gender. The average number of notified cases for men was 213 (standard deviation, SD = 105) and for women was 173 (SD = 86) (*P* = 0.39). From 2014 to 2017, the number of notified cases increased for men (from 80 to 330 cases) and for women (from 57 to 251 cases). The proportion of notified cases was predominated by men over the study period (> 52%), with the male-to-female (M:F) notification ratio consistently > 1.1:1. However, the M:F ratio continuously declined from 1.4 in 2014 to 1.1 in 2016, and then increased to 1.3 in 2017 (Table 1).

#### Trends in case notification stratified by age categories

**Fig. 2:**
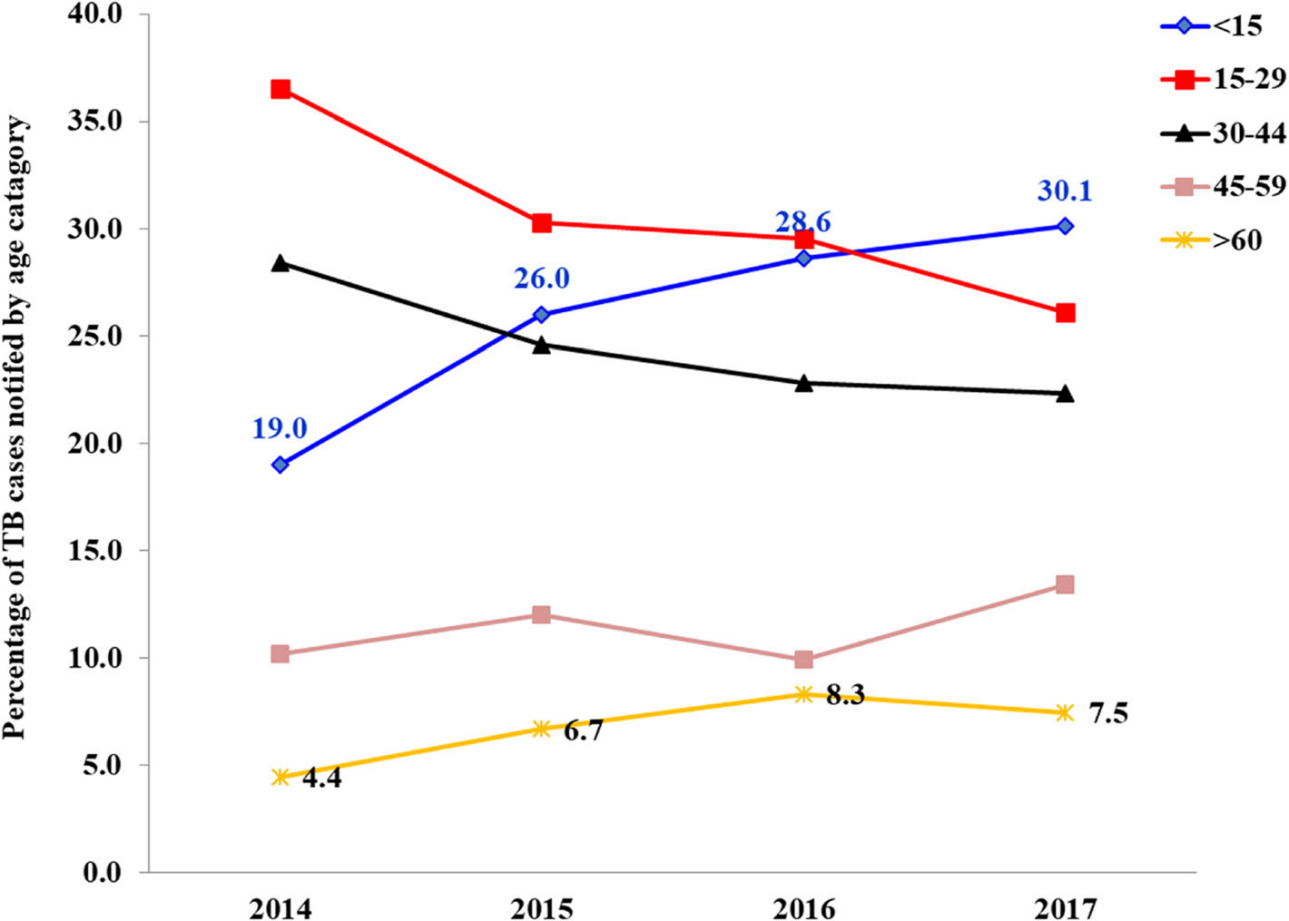
Trends of notified TB case disaggregated by age categories in the refugee camps, Ethiopia, 2014 to 2017

Over the study period, the largest contributor to the total TB cases notified were age 15–29 years followed by 30–44 years. However, from 2014 to 2017, the contribution of age 15–44 years decreased from 64.9 to 48.3%, and that above 45 years gently increased from 4.4 to 7.5%. Although children (< 15 years) were the third largest contributor to the total TB cases in 2014 (19.0%), their share continuously increased over the study period and become the largest contributor by 2017 (30.1%) (Table 1, Fig. 2).

The contribution of children (< 15 years) to EPTB continuously increased and remained at highest level across the years. Of the total EPTB cases notified in 2014, 2015, 2016 and 2017, 40.6, 45.2, 58.2, and 65.1%, respectively, were children under 15 years of age.

#### TB case notification segregated by age group and gender

**Fig. 3:**
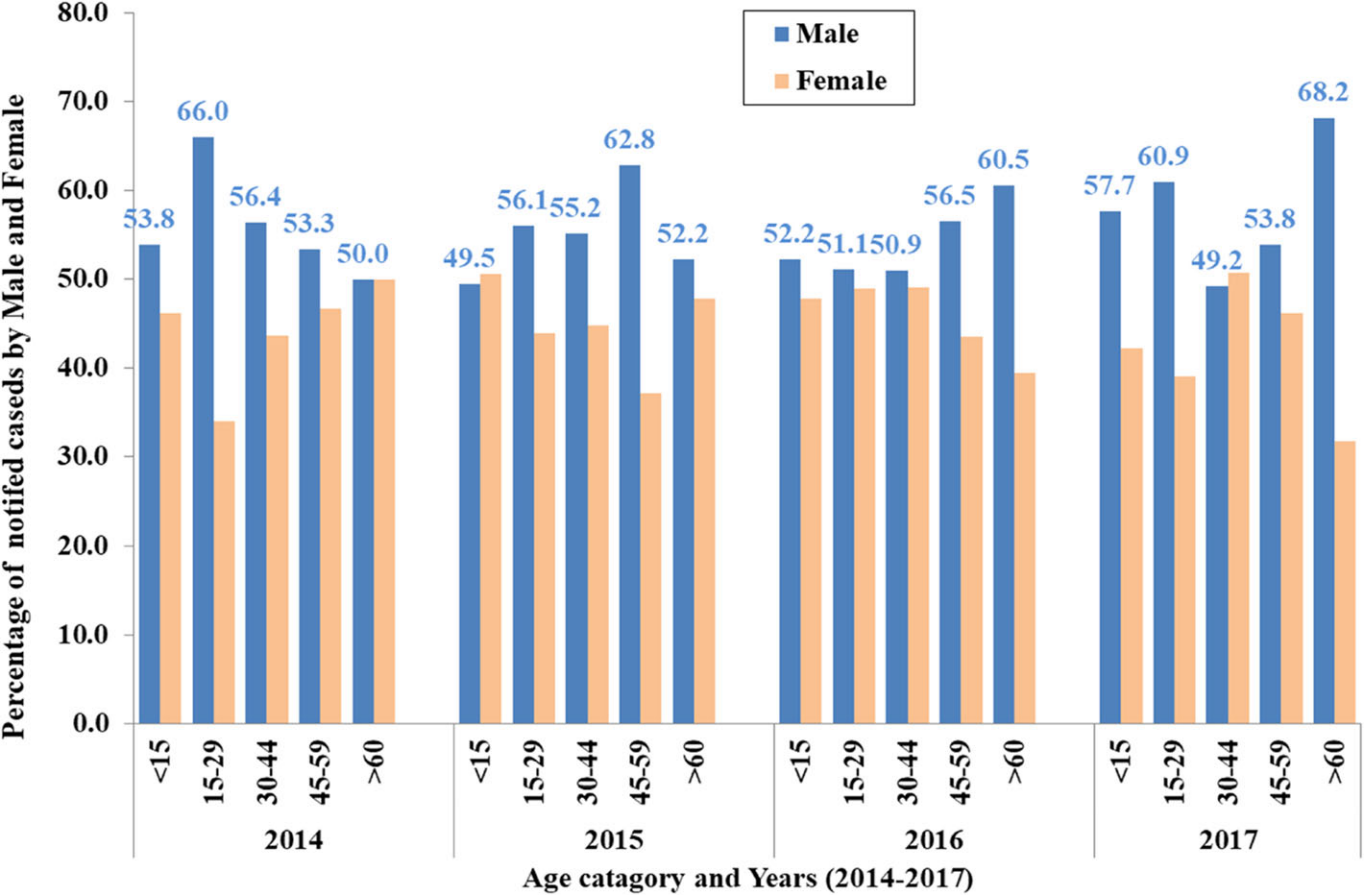
TB cases notified in the refugee camps in Ethiopia by age categories and gender, 2014 to 2017

The percentage of notified TB cases for men was higher (> 50%) than for women in all the age categories (< 15, 15–29, 30–44, 45–59 and > 60 years old) across the years (2014–2017), except for age 60, < 15 and 30–44 years in 2014, 2015 and in 2017, where men constituted 50, 49.5, and 49.2%, respectively (Fig. 3).

### Trends in HIV testing and antiretroviral treatment coverage

HIV testing was performed in 1329 (85.6%) of the 1553 notified cases, among those 165 (12.4%) were HIV positive (Table 1). The percentage of TB patients tested for HIV increased from 84.1% in 2014 to 88.6% in 2017. The percentage of TB patients tested HIV positive was decreased from 15.3% in 2015 to 10.9% in 2017, whereas antiretroviral treatment (ART) coverage increased from 40% in 2014 to 84.2% in 2017 (Table 1).

### Trends in TB treatment outcomes, 2014 to 2017

Trend of treatment outcome was evaluated for a total of 1553 TB cases of all forms registered during the study period (Table 2). Treatment success rate for all TB cases remained lower at a range of 72.4 to 79.4%. On average 24.7% of the patients were cured (increasing from 18.8% in 2014 to 27.7% in 2017) and 50.5% with treatment completed (decreasing from 56.5% in 2014 to 46.6% in 2017). On average 24.8% of the patients had unfavorable treatment outcome, including 11.5% not evaluated, 8.0% LTFU, 4.8% died and 0.5% treatment failed. Of the LTFU cases in 2016 and 2017, 56.1 and 78% % were males, respectively.

**Table 2:** Trends of TB treatment outcomes for TB cases of all forms registered for treatment (*n* = 1553) in refugee camps in Ethiopia, 2014–2017

Treatment outcomes	Years				Total, n%
	2014, n (%)	2015, n (%)	2016, n (%)	2017, n (%)	
Total cases	138	354	473	588	1553
Cured	26 (18.8)	75 (21.2)	119 (25.2)	163 (27.7)	383 (24.7)
completed	78 (56.5)	206 (58.2)	225 (47.5)	274 (46.6)	783 (50.4)
Failed	1 (0.7)	4 (1.1)	1 (0.2)	2 (0.3)	8 (0.5)
LTFU	11 (8.0)	20 (5.6)	42 (8.9)	51 (8.7)	124 (8.0)
Died	5 (3.6)	19 (5.4)	24 (5.1)	26 (4.4)	74 (4.8)
Not evaluated	17 (12.3)	30 (8.5)	62 (13.1)	72 (12.2)	181 (11.7)
***Success rate***	***104 (75.3)***	***281 (79.4)***	***344 (72.7)***	***437 (74.3)***	***1166 (75.1)***

Data are presented as numbers (%)

#### LTFU before treatment initiation

LTFU of smear positive PTB patients before starting treatment will have negative impact on clinical outcome and TB transmission. In the refugee camps, among TB cases who were smear positive PTB and registered in the laboratory log book in 2014, 2015, 2015 and 2017, respectively, 23 (29.1%), 22 (27.9%), 18 (22.8%), and 16 (20.3%) were not registered in the Unit TB register and treatment outcome was not recorded *(LTFU before treatment)*. Of the 16 LTFU before treatment in 2017, all were from Gambella refugee area, where 8 (50.0%) were from Kule, 5 (31.3%) from Terkedi, and 1 (6.2%) from Pugnido Agnewak refugee health facilities.

#### Treatment outcomes by gender, age, and type of TB

**Fig. 4:**
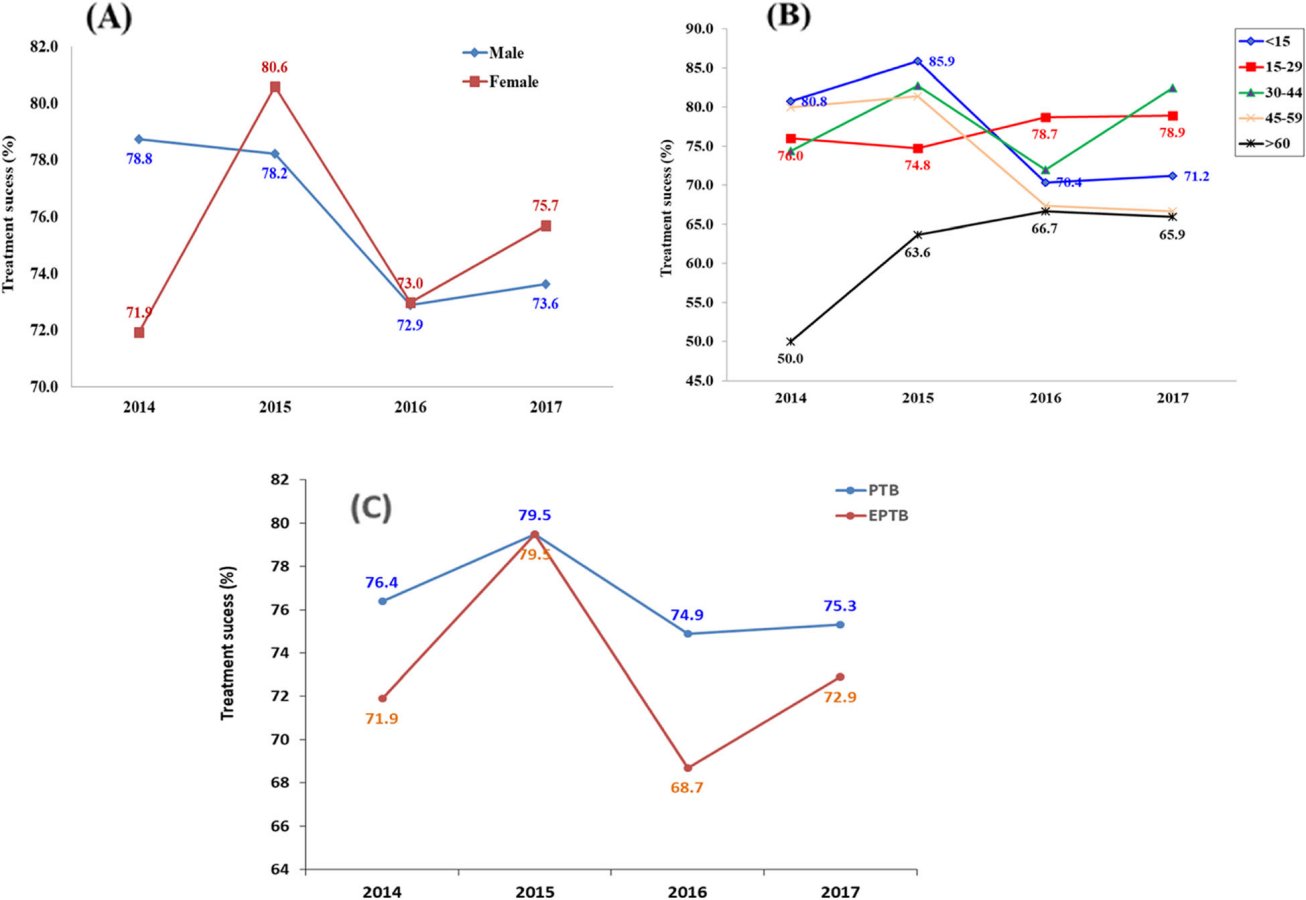
TB treatment success rate of the TB cases registered in the refugee camps in Ethiopia (2014–2017) by gender (**a**), age category (**b**), and by type of TB (**c**)

There was a variation in treatment success rate by gender, age category and type of TB (Fig. 4). From 2015 to 2017, treatment success rate was relatively higher for females and declined for both sexes (from 80.6 to 75.7% for female; from 78.2 to 73.6% for male) (Fig. 4a). Lowest treatment success rate was observed in the oldest age (60+ years old) over the study period, although it increased from 50.0% in 2014 to 65.9% in 2017. The treatment success rate was highest for children (< 15 years) in 2014 (80.8%) and 2015 (85.9%), but declined in 2016 (70.4%) and in 2017 (71.3%). Treatment success rate for the younger age group (15–29 years old) was remained stable (74.8–78.9%) over the study period (Fig. 4b). Treatment success for PTB was higher over the years (except in 2015) than in EPTB, but declined gently from 2015 to 2017 in both groups (Fig. 4c).

### Factors associated with unsuccessful TB treatment outcomes

Based on a multivariable analysis, factors associated with unsuccessful TB treatment outcome (LTFU, failure, and died) were pretreatment weight < 40 Kg (adjusted odds ratio [aOR] = 1.5, 95% confidence interval (CI): 0.9–2.3, *P = 0.05*); age 45–59 years (aOR =2.4, 95% CI:1.3–4.7, *P = 0.008*); age > 60 years (aOR =4.3, 95% CI:2.3–9.1, *P* < 0.001), and HIV infection (aOR = 3.6, 95% CI:2.3–5.8, *P < 0.001*) (Table 3).

**Table 3:** Factors associated with unsuccessful TB treatment outcomes among TB cases of all Forms in refugee camps in Ethiopia (2014–2017)

Characteristics	Successful outcomes	Unsuccessful outcomes	X^2^, *p*-value	Bivariate analysis		Multivariable analysis	
				OR (95%CI)	***P*** value	Adjusted OR (95% CI)	***P*** value
Gender							
Male	637 (84.3)	119 (15.7)	0.80;	1			
Female	522 (86.0)	85 (14.0)	0.37	1.04 (0.8–1.3)	0.74		
**Age group**							
< 15	321 (86.5)	50 (13.5)		1			
15–29	349 (87.0)	52 (13.0)	**33.7;**	0.9 (0.6–1.4)	0.8		
30–44	286 (89.4)	34 (10.6)	**< 0.001**	0.8 (0.5–1.2)	0.3		
45–59	130 (77.8)	37 (22.16)		1.8 (1.1–2.9)	**0.01**	2.4 (1.3–4.7)	**0.008**
> = 60	72 (69.2)	32 (30.8)		2.8 (1.7–4.8)	**0.001**	4.3 (2.3–9.1)	**< 0.001**
Pre-treatment weight, kg							
> 40	576 (86.9)	87 (13.1)	**6.0;**	1	**0.08**		
< 40	215 (80.5)	52 (19.5)	**0.01**	1.3 (0.9–1.8)		1.5 (0.9–2.3)	**0.05**
**Type of TB**							
P/Neg	314 (82.6)	66 (17.4)	**6.5;**	1			
PTB+	528 (87.9)	73 (12.1)	**0.09**	0.6 (0.4–0.9)	**0.02**	0.6 (0.4–1.0)	**0.06**
Smear not done	4 (80.0)	1 (20.0)		1.2 (0.1–10.8)	0.8		
EPTB	313 (83.2)	63 (16.8)		0.9 (0.6–1.4)	0.8		
**Category of TB patients**							
New and Relapse	1146 (85.0	202 (15.0)		1			
Failure	2 (100)	0	**1.9;**	2.1 (0.6–8.1)	0.2		
LTFU	8 (72.7)	3 (27.3)	**0.6**	1.8 (0.2–18.3)	0.6		
Others(O)	3 (75.0)	1 (25.0)					
**HIV status**							
Negative	934 (87.8)	130 (12.2)	**24.6;**	1	**< 0.001**		
Positive	102 (72.3)	39 (27.7)	**< 0.001**	1.6 (1.3–2.0)		3.6 (2.3–5.8)	**< 0.001**
**ART initiated?**^**a**^							
Yes	89 (74.8)	30 (25.2)	**3.5;**	1	**0.06**		
No	148 (83.6)	29 (16.4)	**0.06**	0.9 (0.3–1.0)			

^a^Odds ratio (OR) (95%CI) are based on 268 HIV positive study participants

X^2 =^ chi square

*P* value < 0.05 was considered statistically significant

## Discussion

This study provides evidences on the indicators of TB program performance that can help to improve the effectiveness of the TB control and prevention in the refugee camps in Ethiopia. During the study period, TB case notification was continuously increasing, and TB treatment success rate remained lower than the national achievement and the global target. TB patients with pretreatment weight below 40 Kg, age over 45 years and HIV positives are at higher risk for unsuccessful TB treatment outcomes.

In principle, in settings with high-performance of TB surveillance system (less under diagnosis and underreporting of TB cases) TB case notification provides a good proxy indication for TB incidence [2]. In the refugee camps, the number of notified TB cases continuously increased from 138 in 2014 to 588 cases in 2017. Similarly, a study done in the refugee population in Gambella Region of Ethiopia, showed 29.0% increase in notified TB cases in 8 years (2009–2017) [8].

Although needs further investigation, there are four possible reasons that can explain the increased trend in TB case notification in the refugee camps: 1) real increase in TB incidence due to problems in the health care system that negatively affects the performance of TB programs, 2) surrounding community level factors that enhance TB transmission, 4) improvement in case detection, case notification and recording and reporting system, and 3) an increase in number of refugee population overtime (from 138 in 2014 to 588 in 2017). In support to our statement, continuous increase in case notification [13] and in case detection rate [14] has been reported in the Syrian refugees in Lebanon and Jordan, respectively, following the continuous immigration of Syrian refugees to both countries. In addition, an association between immigration and increased prevalence of either TB or EPTB has been reported [12]. Together, our findings suggest the need to strengthen TB prevention and control strategies in the refugee camps in Ethiopia.

Evidence on TB epidemiology by type of TB (PTB and EPTB) will help to implement targeted TB diagnostic, treatment and prevention services. EPTB represented 32.7% of the total cases notified in the refugee camps in 2017. This is similar to the 31% EPTB cases among notified TB cases in Ethiopia in 2017 [19], but higher than the 15% EPTB among incident TB cases in the globe in 2018 [2]. However, there was increased trend in the proportion of EPTB in the refugee camps from 2014 (23.2%) to 2017 (32.7%). Together, our findings indicate a change in the epidemiology of EPTB in the refugee camps overtime, which suggests the need of further investigation to assess the possible confounding factors and to design targeted diagnostic, treatment and prevention strategies.

Evidences on the status of bacteriologically confirmed PTB, which is known as infectious TB, is essential to monitor resistance, disease severity, treatment response, and spread of TB. Limited access to health facilities and to diagnostic services, and low treatment success could contribute to high rate of TB transmissions and to higher smear positive PTB. Of the new and relapse pulmonary cases in 2017 in the refugee camps, 64.8% were bacteriologically confirmed, which is higher than that for Ethiopia (58%) and for the globe (56%) as reported by WHIO 2018 [2]. Nonetheless, the proportion of bacteriologically confirmed pulmonary new and relapse cases in the refugee camps increased from 43.8% in 2014 to 64.8% in 2017. We speculate, this could be due to expansion of diagnostic services and an increase in the number and better utilization of bacteriologic diagnostics. However, the low proportion of bacteriological confirmed cases by 2017 (66.2%) can further be improved by refresher training and introduction and expansions of molecular diagnostics (sputum smear, molecular and culture).

TB affects both sexes but disproportionally males [2]. Women face barriers to TB diagnosis and are less likely to report or show evidence of typical symptoms of pulmonary TB (cough, sputum production, and haemoptysis) [20, 21]. Despite that more than 70% of the refugees in the camps are women and children, males accounted 54.7% of the total notified TB cases in this study. Similarly, 64% of the global and 57.3% of the national (Ethiopia) TB incidences in 2018 were male [2]. Other studies in Ethiopia also showed > 50% of the notified TB cases to be males [22, 23]. The M:F ratio for notification in the refugee camps was > 1.1:1 across the study period.

TB affects all age groups but disproportionally. According to 2018 WHO report, people in the age group 15–24 years in the globe and 15–34 years in Ethiopia are disproportionately affected by TB [2]. Similarly, in the refugee camps investigated in the current study, TB patients in the age groups 15–44 years represented 52.5% of the notified TB cases. Therefore, as people in this age group represents an active component of the workforce and have major impact on the socioeconomic of the society, the refugee TB programs and partners should tailor interventions and case finding efforts focusing on the age group 15 to 44 years.

Pediatric TB can has been considered as a sentinel for TB transmission. During this study period, the contribution of children (< 15 years) to the total TB cases notified and to EPTB increased from 18.8 to 30.1%, and from 40.6 to 65.1%, respectively. Globally, 10% of the TB incidence in 2018 were children (aged < 15 years) [2]. Although need further investigation, we speculate the increase in childhood TB could be due to real increase in TB transmission and incidence, an increase in number of children among refugees, and introduction of improved diagnostics like Gene Xpert.

HIV co-morbidity is one of the most important risk factors for TB. ART on the other hand plays significant role in reducing TB related morbidity and mortally, and TB incidence [24]. Across the study period, highest HIV prevalence (13.9–21.6%) among the TB patient was observed in Gambella, followed by Shire and Jijiga areas. Previous study in Gambella Region of Ethiopia showed 31.2% HIV co-infection among refugee TB patients [21]. Among the HIV positive TB patients in the refugee camps in the current study, 84.2% were on ART in 2017. This is lower as compared to the 92% HIV positive TB patients in Ethiopia who were on ART by 2017 [2]. These evidences suggest the need to strengthen TB/HIV collaborative activities in the refugee camps with special focus to Gambella, Shire and Jijiga refugee areas.

The priorities of a TB programme are to identify and treat infectious TB patients with smear-positive PTB and those with severe forms of the disease. Thus, cure of infectious patients is the most effective means of reducing TB transmission in the family and community. In the refugee camps, although the percentage of cured patients continuously increased during the study period only 27.7% of the patients by 2017 were cured, which is comparable to the 28.5% cure rate for TB patients registered in 722 districts for the period 2015–2017 in Ethiopia [25], and extremely lower than the 85% threshold defined by WHO [2]. This lower proportion of cured patients in the refugee camps can be increased by improving follow-up sputum smear and culture examination, recording and reporting system, refresher training to health professionals, and engagement of patients, care givers and communities.

Although deceased overtime (from 56.5% in 2014 to 46.6% in 2017), on average 50.4% of the patients in this study had treatment completed (either follow up sputum smear is not done or results are not recorded) which is lower than the 62.4% treatment completed for TB patients registered in 722 districts for the period 2015–2017 in Ethiopia [25]. These results indicate the gap in performing follow up sputum smear examination, patient monitoring and in recording and reporting system in the refugee camps which need interventional actions.

According to WHO 90-(90)-90 global targets (which should be reached ideally by 2020 and at the latest by 2025), at least 90% TB treatment success rate need to be achieved among people on TB treatment in order to end TB by 2035 [2]. In this study, despite the increased trend in notified TB cases, treatment success rate for all TB cases remained lower and stable at a range of 72.4 to 79.4%. This is lower than the 90% global target [2]; the global (85%) and national/Ethiopia (96.0%) treatment success rate for new and relapse cases in 2017 [2]; and the 94% treatment success for bacteriologically confirmed PTB cases in Ethiopia in 2016/17 [19]. However, the mean treatment success rate in the refugee camps (75.1%) was higher than that reported in refugee camps in Syrian (63.6%) [16], in Gambella, Ethiopia (74.2%) [19], and other refugee camps in different part of the world (66.5 to 77.5%) [26–29].

The lower treatment success rate in Ethiopia refugee camps (average 75.1%) was attributed to higher “not evaluated” (11.5%), LTFU (8.0%), and Died (4.8%) treatment outcomes. Other studies conducted in Ethiopia showed 8.5–18.3% LTFU [23, 30, 31] and 0.2–7.8% treatment failure [23, 32]. Together, the higher unfavorable treatment outcomes (LTFU, death, not evaluated) in the refugee camps can be addressed by improving adherence education, regular monitoring and evaluation, regular communication and collaboration between health professionals, patients and community mobilizers in defaulter tracing.

Interestingly, of the sputum smear positive cases in 2017, 16 (20.3%) were not registered in the Lab and Unit TB register *(LTFU before treatment).* Majority of the LTFU before treatment patients are in Gambella refugee area. This could be due to the movement of the refugees which are originated from South Sudan. Therefore, since this LTFU before treatment could have significant negative impact on the clinical outcome of the patients as well as on TB transmission, special patient monitoring and support is recommend in Gambella refugee area.

Understanding factors associated with unsuccessful treatment outcomes can help to design appropriate interventions. In this study pretreatment weight < 40 kg, older age (> 45 years), and HIV infection were independently associated with unsuccessful treatment outcomes. Independent association of older age (> 30 years [33], retreatment and HIV co-infected [31] with unfavorable treatment outcome was also reported by others. Thus, TB patients with pretreatment weight < 40 kg, older age (> 45 years), and HIV infected need special social and economic support, early diagnosis and close monitoring throughout their treatment period in the refugee camps in Ethiopia.

The positive association of the age group > 45 years with unsuccessful treatment outcome could be that this age group have a higher tendency not to adhere to anti-TB treatment because of tightness with work, travel a long distance to search work and addicted to alcohol [34]. The positive association of HIV infection with unsuccessful treatment outcome could be due less adherence of HIV patients to TB treatment due to drug burden or it could be due to less drug absorption related to due to drug-drug interaction.

### Our analysis has some limitations

Firstly, since this study was conducted retrospectively using secondary data, data completeness could be an issue. Thus, some important socioeconomic and clinical data of the TB patients and health facility level data could be missed (not documented or TB register not contain information). However, maximum effort has been done to maintain the data quality standards including training of data collectors and supervisors, daily supervision, 10% data re-entry at the field, and data verification during data entry and analysis. Secondly, the use of secondary data in this retrospective study did not permit us to analyse patient socioeconomic (education, poverty, living condition) and health facility level factors (access and use) that may be associated with unsuccessful treatment outcomes.

### The strengths of the study

We have provided useful information on the performance of TB program (case notification and treatment outcome, and factors associated with unsuccessful treatment outcomes) in seven refugee areas and 25 refugee camps over 4 year timespan in Ethiopia that will help to guide future TB control efforts in refugee camps.

## Conclusions

This study provided useful evidence which will help to improve the effectiveness of TB programs in the refugee camps. The number of notified TB cases, percentage of EPTB, and proportion of bacteriologically confirmed pulmonary new and relapse cases increased from 2014 to 2017. The contribution of children (< 15 years) to the total notified TB cases and to EPTB increased over the study period. Men and age groups 15–44 represented highest share of the notified TB cases. TB treatment success rate remained far lower below the global target (90%) and needs to be improved. The higher LTFU, not evaluated and died treatment outcomes can be improved by intensifying adherence education patient monitoring. Special socioeconomic support and close monitoring is recommended for TB patients who are at risk for unsuccessful treatment (pretreatment weight < 40 kg, > 45 years, and HIV infected). We recommend future study to investigate the reasons for lower treatment success, and increased trend in case notification, childhood TB and bacteriologically confirmed new and relapse.

## Supplementary Information

**Additional file 1:.** Distribution of the notified TB case in the seven refugee areas and 25 refugee health facilities/camps in Ethiopia (2014–2017).

**Additional file 2:.** Definitions.

## Data Availability

All data generated or analysed during this study are included in this published article.

## Abbreviations

aOR: Adjusted odds ratio
ART: Antiretroviral treatment
ARRA: Agency for refugee and returnee Affair
DP: Displaced populations
EPTB: Extrapulmonary PTB
HIV: Human Immunodeficiency virus
LTFU: Lost-to-follow up
MDR: Multi-drug resistant
MTB: *Mycobacterium tuberculosis*
PTB: Pulmonary TB
PTB +: Smear positive pulmonary TB.
P/Neg: smear negative pulmonary TB
TB: Tuberculosis
WHO: World health organization
