# Supplementary material for: Tuberculosis (TB) in the refugee camps in Ethiopia: trends of case notification, profile, and treatment outcomes, 2014 to 2017
Source: BMC Infect Dis. 2021 Feb 3;21:139. doi: 10.1186/s12879-021-05828-y (PMC7856765; doi:10.1186/s12879-021-05828-y)
Supplement: Supplementary file 1 — Additional file 1:. Distribution of the notified TB case in the seven refugee areas and 25 refugee health facilities/camps in Ethiopia (2014–2017). [file 12879_2021_5828_MOESM1_ESM.docx]

Supplement 1: Distribution of the notified TB case in the seven refugee areas and 25 refugee health facilities/camps in Ethiopia (2014–2017)

| Regional state | Refugee areas | Refugee Health facilities/ camps | Number of TB cases /year | | | |  |
| --- | --- | --- | --- | --- | --- | --- | --- |
|  |  |  | 2014 | 2015 | 2016 | 2017 | Total |
| TIgray | 1. Shire | 1.      Mai-Aini | 10 | 4 | 8 | 8 | 30 |
|  |  | 2.      Adi Harush | 8 | 10 | 4 | 7 | 29 |
|  |  | 3.      Shimelba | 3 | 12 | 8 | 6 | 29 |
|  |  | 4.      Hitsats | 0 | 2 | 6 | 7 | 15 |
|  |  | Total | 21 | 28 | 26 | 28 | 103 |
| Afar | 2. Semera | 5.      Aysaita | 0 | 0 | 4 | 40 | 44 |
|  |  | 6.      Barahle | 0 | 0 | 2 | 7 | 9 |
|  |  | Total | 0 | 0 | 6 | 47 | 53 |
| Gambella | 3. Gambella | 7.      Pugnido-New site | 0 | 16 | 44 | 27 | 87 |
|  |  | 8.      Pugnido-Agnewak | 10 | 27 | 28 | 26 | 91 |
|  |  | 9.      Pugnido-Village 12 | 15 | 23 | 16 | 18 | 72 |
|  |  | 10.   Kule | 0 | 27 | 124 | 183 | 334 |
|  |  | 11.   Jewi | 0 | 0 | 34 | 34 | 68 |
|  |  | 12.   Tierkidi | 18 | 66 | 42 | 80 | 206 |
|  |  | Total | 43 | 159 | 288 | 368 | 858 |
| South nations and nationalities | 4. Mizan | 13.   Okugo | 2 | 30 | 18 | 29 | 79 |
| Asossa | 5. Asossa | 14.   Sherkole | 10 | 9 | 4 | 4 | 27 |
|  |  | 15.   Bambasi | 4 | 6 | 7 | 6 | 23 |
|  |  | 16.   Tongo | 0 | 11 | 3 | 3 | 17 |
|  |  | 17.   Tsore | 0 | 3 | 2 | 1 | 6 |
|  |  | Total | 14 | 29 | 16 | 14 | 73 |
| Somalia | 6. Jijiga | 18.   Kebribeya | 19 | 18 | 19 | 17 | 73 |
|  |  | 19.   Aw-barre | 10 | 8 | 11 | 6 | 35 |
|  |  | 20.   Sheder | 16 | 16 | 15 | 11 | 58 |
|  |  | Total | 45 | 42 | 45 | 34 | 166 |
| Somalia | 7. Dollo Ado | 21.   Melkadida | 13 | 23 | 20 | 18 | 74 |
|  |  | 22.   Kobe | 0 | 12 | 13 | 16 | 41 |
|  |  | 23.   Hilaweyn | 0 | 13 | 16 | 15 | 44 |
|  |  | 24.   Buramino | 0 | 18 | 11 | 4 | 33 |
|  |  | 25.   Bokolmayo | 0 | 0 | 14 | 15 | 29 |
|  |  | Total | 13 | 66 | 74 | 68 | 221 |
