# Supplementary material for: Tuberculosis (TB) in the refugee camps in Ethiopia: trends of case notification, profile, and treatment outcomes, 2014 to 2017
Source: BMC Infect Dis. 2021 Feb 3;21:139. doi: 10.1186/s12879-021-05828-y (PMC7856765; doi:10.1186/s12879-021-05828-y)
Supplement: Supplementary file 2 — Additional file 2:. Definitions. [file 12879_2021_5828_MOESM2_ESM.docx]

## Supplement 2: Definitions

- **TB case notification:** is where TB is diagnosed in a patient and is reported within the national TB surveillance system.
- **TB cases:** is a patient in whom TB has been diagnosed**.**
- **A bacteriologically confirmed TB case**: is one from whom a biological specimen is positive by smear microscopy, culture or WHO approved rapid diagnostics (such as Xpert MTB/RIF); and
- **Clinically diagnosed TB case**: is one who does not fulfil the criteria for bacteriological confirmation but has been diagnosed with active TB by a clinician or other medical practitioner who has decided to give the patient a full course of TB treatment.
- **Extra pulmonary tuberculosis (EPTB):** any bacteriologically confirmed or clinically diagnosed **case of TB** involving organs other than the lungs; and
- **Pulmonary tuberculosis (PTB):** any bacteriologically confirmed or clinically diagnosed **case of TB** involving the lung parenchyma or the tracheobronchial tree.
  - **Pulmonary TB positive (PTB+):** Bacteriologically confirmed pulmonary TB cases using available confirmatory diagnostic methods; and
  - **Pulmonary TB negative (P/Neg):** Clinically diagnosed pulmonary TB cases.

**Patients were classified into two groups according to their TB treatment history**:

1. **New TB patients:** are those who have never been treated for TB or have taken anti-TB drugs for less than 1 month;
2. **Previously treated TB patients:** are those who have received one month or more of anti-TB drugs in the past, and were categorized further as:

**Relapse patients:** are those who have previously been treated for TB, were declared cured or treatment completed at the end of their most recent course of treatment, and are now diagnosed with a recurrent episode of TB (either a true relapse or a new TB caused by reinfection);

**Treatment after failure patients:** are those who have previously been treated for TB and whose treatment failed at the end of their most recent course of treatment;

**Treatment after loss to follow-up patients:** are those who have previously been treated for TB and were declared lost to follow-up at the end of their most recent course of treatment. *(These were previously known as treatment after default patients);*

**Other previously treated patients:** are those who have previously been treated for TB but whose outcome after their most recent course of treatment is unknown or undocumented;

**Transfer in:** A patient who started treatment in one health facility (reporting unit) and transferred to another health facility (reporting unit) to continue treatment.

- **Treatment outcomes were categorised as follows**:

***Cured:*** A pulmonary TB patient with bacteriologically confirmed TB at the beginning of treatment who was smear- or culture-negative in the last month of treatment and on at least one previous occasion;

***Treatment Completed:*** A TB patient who completed treatment without evidence of failure **BUT** with no record to show that sputum smear or culture results in the last month of treatment and on at least one previous occasion were negative either because tests were not done or because results are unavailable;

***Treatment failed:*** A TB patient whose sputum smear or culture is positive at month 5 **or** later during treatment;

***Lost to follow up:*** A TB patient who did not start treatment **or** whose treatment was interrupted for 2 consecutive months or more;

***Died:*** A TB patient who dies for any reason before starting **or** during the course of treatment;

***Not evaluated:*** A TB patient for whom no treatment outcome is assigned. This includes cases “transferred out” to another treatment unit as well as cases for whom the treatment outcome is unknown to the reporting unit.

**Successful treatment outcome:** cured and treatment completed) and ;

**Unsuccessful treatment outcome:** died, treatment failed and lost to follow up.
